# Supplementary material for: Iron Content of Wheat and Rice in Australia: A Scoping Review
Source: Foods. 2024 Feb 10;13(4):547. doi: 10.3390/foods13040547 (PMC10888283; doi:10.3390/foods13040547)
Supplement: Supplementary file 1 [file foods-13-00547-s001.zip › foods-2813256-supplementary.pdf]

PRISMA 2020 Checklist

Section and Topic	Item #	Checklist item	Location where item is reported
**TITLE**			
Title	1	Identify the report as a systematic review.	P 1
**ABSTRACT**			
Abstract	2	See the PRISMA 2020 for Abstracts checklist.	P 1
**INTRODUCTION**			
Rationale	3	Describe the rationale for the review in the context of existing knowledge.	P 2
Objectives	4	Provide an explicit statement of the objective(s) or question(s) the review addresses.	P 2
**METHODS**			
Eligibility criteria	5	Specify the inclusion and exclusion criteria for the review and how studies were grouped for the syntheses.	P 2
Information sources	6	Specify all databases, registers, websites, organisations, reference lists and other sources searched or consulted to identify studies. Specify the date when each source was last searched or consulted.	P2-3
Search strategy	7	Present the full search strategies for all databases, registers and websites, including any filters and limits used.	
Selection process	8	Specify the methods used to decide whether a study met the inclusion criteria of the review, including how many reviewers screened each record and each report retrieved, whether they worked independently, and if applicable, details of automation tools used in the process.	P 3
Data collection process	9	Specify the methods used to collect data from reports, including how many reviewers collected data from each report, whether they worked independently, any processes for obtaining or confirming data from study investigators, and if applicable, details of automation tools used in the process.	P 3
Data items	10a	List and define all outcomes for which data were sought. Specify whether all results that were compatible with each outcome domain in each study were sought (e.g. for all measures, time points, analyses), and if not, the methods used to decide which results to collect.	P 3
	10b	List and define all other variables for which data were sought (e.g. participant and intervention characteristics, funding sources). Describe any assumptions made about any missing or unclear information.	P 3
Study risk of bias assessment	11	Specify the methods used to assess risk of bias in the included studies, including details of the tool(s) used, how many reviewers assessed each study and whether they worked independently, and if applicable, details of automation tools used in the process.	n/a
Effect measures	12	Specify for each outcome the effect measure(s) (e.g. risk ratio, mean difference) used in the synthesis or presentation of results.	n/a
Synthesis methods	13a	Describe the processes used to decide which studies were eligible for each synthesis (e.g. tabulating the study intervention characteristics and comparing against the planned groups for each synthesis (item #5)).	P 3
	13b	Describe any methods required to prepare the data for presentation or synthesis, such as handling of missing summary statistics, or data conversions.	P 3
	13c	Describe any methods used to tabulate or visually display results of individual studies and syntheses.	P 3
	13d	Describe any methods used to synthesize results and provide a rationale for the choice(s). If meta-analysis was performed, describe the model(s), method(s) to identify the presence and extent of statistical heterogeneity, and software package(s) used.	P 3
	13e	Describe any methods used to explore possible causes of heterogeneity among study results (e.g. subgroup analysis, meta-regression).	n/a
	13f	Describe any sensitivity analyses conducted to assess robustness of the synthesized results.	n/a
Reporting bias assessment	14	Describe any methods used to assess risk of bias due to missing results in a synthesis (arising from reporting biases).	n/a

Supplementary Table S1: PRISMA 2020 Checklist

Section and Topic	Item #	Checklist item	Location where item is reported
Certainty assessment	15	Describe any methods used to assess certainty (or confidence) in the body of evidence for an outcome.	
**RESULTS**			
Study selection	16a	Describe the results of the search and selection process, from the number of records identified in the search to the number of studies included in the review, ideally using a flow diagram.	P 3-4
	16b	Cite studies that might appear to meet the inclusion criteria, but which were excluded, and explain why they were excluded.	P 3-4
Study characteristics	17	Cite each included study and present its characteristics.	P 4-5
Risk of bias in studies	18	Present assessments of risk of bias for each included study.	n/a
Results of individual studies	19	For all outcomes, present, for each study: (a) summary statistics for each group (where appropriate) and (b) an effect estimate and its precision (e.g. confidence/credible interval), ideally using structured tables or plots.	P 6-10
Results of syntheses	20a	For each synthesis, briefly summarise the characteristics and risk of bias among contributing studies.	P 5
	20b	Present results of all statistical syntheses conducted. If meta-analysis was done, present for each the summary estimate and its precision (e.g. confidence/credible interval) and measures of statistical heterogeneity. If comparing groups, describe the direction of the effect.	P 11
	20c	Present results of all investigations of possible causes of heterogeneity among study results.	P 11
	20d	Present results of all sensitivity analyses conducted to assess the robustness of the synthesized results.	n/a
Reporting biases	21	Present assessments of risk of bias due to missing results (arising from reporting biases) for each synthesis assessed.	n/a
Certainty of evidence	22	Present assessments of certainty (or confidence) in the body of evidence for each outcome assessed.	n/a
**DISCUSSION**			
Discussion	23a	Provide a general interpretation of the results in the context of other evidence.	P 12-14
	23b	Discuss any limitations of the evidence included in the review.	P 14
	23c	Discuss any limitations of the review processes used.	P 14
	23d	Discuss implications of the results for practice, policy, and future research.	P 14
**OTHER INFORMATION**			
Registration and protocol	24a	Provide registration information for the review, including register name and registration number, or state that the review was not registered.	n/r
	24b	Indicate where the review protocol can be accessed, or state that a protocol was not prepared.	n/r
	24c	Describe and explain any amendments to information provided at registration or in the protocol.	n/a
Support	25	Describe sources of financial or non-financial support for the review, and the role of the funders or sponsors in the review.	P 14
Competing interests	26	Declare any competing interests of review authors.	P 14
Availability of data, code and other materials	27	Report which of the following are publicly available and where they can be found: template data collection forms; data extracted from included studies; data used for all analyses; analytic code; any other materials used in the review.	P 14

From: Page MJ, McKenzie JE, Bossuyt PM, et al. The PRISMA 2020 statement: an updated guideline for reporting systematic reviews. *BMJ* 2021;372:n71. doi:10.1136/bmj.n71

For more information, visit: **www.prisma-statement.org**.

Search Strategy

Search Strategy: Search terms used on Embase (OVID) for finding literature on iron content in wheat and rice, which was executed on the 21st of August 2023.

1. wheat/ 41965
2. wheat*.mp. 81175
3. common wheat.mp. 1686
4. triticum/ 34773
5. triticum aestivum.mp. 10949
6. triticum aestivum L.mp. 5252
7. triticum spelta.mp. 75
8. triticum durum.mp. 957
9. triticum dicoccon.mp. 14
10. triticum dicoccum.mp. 63
11. triticum turgidum.mp. 703
12. triticum turgidum ssp turanicum.mp. 5
13. spelt*.mp. 1550
14. durum*.mp. 3115
15. emmer*.mp. 737
16. wild emmer*.mp. 227
17. einkorn*.mp. 183
18. rice*.mp. 90103
19. rice/ 41618
20. "rice bran".mp. 3120
21. "brown rice".mp. 1724
22. japonica.mp. 17561
23. grain*.mp. 100225
24. grain/ 19533
25. "Oryza sativa subsp. japonica".mp. 47
26. "Japonica rice".mp. 1077
27. "wild rice".mp. 846
28. 1 or 2 or 3 or 4 or 5 or 6 or 7 or 8 or 9 or 10 or 11 or 12 or 13 or 14 or 15 or 16 or 17 or 18 or 19 or 20 or 21 or 22 or 23 or 24 or 25 or 26 or 27 261417
29. iron, dietary/ 5803
30. iron.mp. 394637
31. Iron/ 207865
32. Fe.mp. 144058
33. Iron Intake/ 5803
34. ((mineral or nutri*) adj2 (content or composition or concentration or level* or value or density)).tw. 117328

35. (iron adj2 diet*).mp. 5108
36. ((iron or fe) adj3 (content or composition)).tw. 11142
37. micronutrient/ 40382
38. micronutrient*.tw. 26524
39. trace element/ 42204
40. "trace element".tw. 12546
41. ((physiochemical or chemical or nutri*) adj2 (propert* or parameter*)).tw. 55182
42. mineral/ 53716
43. mineral*.mp. 320053
44. mineral densit*.mp. 73232
45. mineral concentra*.mp. 1297
46. mineral composition*.mp. 2649
47. mineral content*.mp. 15392
48. nutrient/ 53291
49. nutrient content*.mp. 10068
50. nutrient densit*.mp. 1320
51. nutrient concentra*.mp. 5906
52. nutrient composition*.mp. 2636
53. nutritive value/ 24681
54. inorganic nutrient/ 532
55. food composition/ 22269
56. transition element/ 18856
57. mineral intake/ 2205
58. iron content/ 25
59. ferritin/ or ferritin.mp.78771
60. 29 or 30 or 31 or 32 or 33 or 34 or 35 or 36 or 37 or 38 or 39 or 40 or 41 or 42 or 43 or 44 or 45 or 46 or 47 or 48 or 49 or 50 or 51 or 52 or 53 or 54 or 55 or 56 or 57 or 58 or 59 983852
61. "Australia and New Zealand"/ or Australia/ 199789
62. New South Wales/ 4519
63. Queensland/ 4726
64. Victoria/ 3628
65. Australian Capital Territory/ 330
66. Northern Territory/ 663
67. South Australia/ 1934
68. Tasmania/ 914
69. Western Australia/ 3273
70. Sydney.mp. [mp=title, abstract, heading word, drug trade name, original title, device manufacturer, drug manufacturer, device trade name, keyword heading word, floating subheading word, candidate term word] 18388
71. Brisbane.mp. [mp=title, abstract, heading word, drug trade name, original title, device manufacturer, drug manufacturer, device trade name, keyword heading word, floating subheading word, candidate term word] 5242

72. Melbourne.mp. [mp=title, abstract, heading word, drug trade name, original title, device manufacturer, drug manufacturer, device trade name, keyword heading word, floating subheading word, candidate term word] 14525
73. Canberra.mp. [mp=title, abstract, heading word, drug trade name, original title, device manufacturer, drug manufacturer, device trade name, keyword heading word, floating subheading word, candidate term word] 1430
74. Darwin.mp. [mp=title, abstract, heading word, drug trade name, original title, device manufacturer, drug manufacturer, device trade name, keyword heading word, floating subheading word, candidate term word] 3451
75. Adelaide.mp. [mp=title, abstract, heading word, drug trade name, original title, device manufacturer, drug manufacturer, device trade name, keyword heading word, floating subheading word, candidate term word] 4254
76. Hobart.mp. [mp=title, abstract, heading word, drug trade name, original title, device manufacturer, drug manufacturer, device trade name, keyword heading word, floating subheading word, candidate term word] 506
77. Perth.mp. [mp=title, abstract, heading word, drug trade name, original title, device manufacturer, drug manufacturer, device trade name, keyword heading word, floating subheading word, candidate term word] 3797
78. NSW.mp. [mp=title, abstract, heading word, drug trade name, original title, device manufacturer, drug manufacturer, device trade name, keyword heading word, floating subheading word, candidate term word] 8059
79. QLD.mp. [mp=title, abstract, heading word, drug trade name, original title, device manufacturer, drug manufacturer, device trade name, keyword heading word, floating subheading word, candidate term word] 607
80. VIC.mp. [mp=title, abstract, heading word, drug trade name, original title, device manufacturer, drug manufacturer, device trade name, keyword heading word, floating subheading word, candidate term word] 2394
81. ACT.mp. [mp=title, abstract, heading word, drug trade name, original title, device manufacturer, drug manufacturer, device trade name, keyword heading word, floating subheading word, candidate term word] 414337
82. NT.mp. [mp=title, abstract, heading word, drug trade name, original title, device manufacturer, drug manufacturer, device trade name, keyword heading word, floating subheading word, candidate term word] 71527
83. SA.mp. [mp=title, abstract, heading word, drug trade name, original title, device manufacturer, drug manufacturer, device trade name, keyword heading word, floating subheading word, candidate term word] 77273
84. WA.mp. [mp=title, abstract, heading word, drug trade name, original title, device manufacturer, drug manufacturer, device trade name, keyword heading word, floating subheading word, candidate term word] 13339
85. TAS.mp. [mp=title, abstract, heading word, drug trade name, original title, device manufacturer, drug manufacturer, device trade name, keyword heading word, floating subheading word, candidate term word] 11255

86. Oceania.mp. [mp=title, abstract, heading word, drug trade name, original title, device manufacturer, drug manufacturer, device trade name, keyword heading word, floating subheading word, candidate term word] 3143
87. Oceanic Region.mp. [mp=title, abstract, heading word, drug trade name, original title, device manufacturer, drug manufacturer, device trade name, keyword heading word, floating subheading word, candidate term word] 69
88. exp Australia/ 199922
89. Australia*.mp.320589
90. oceanic*.mp. 12272
91. pacific island.mp. or Pacific islands/ 8929
92. 61 or 62 or 63 or 64 or 65 or 66 or 67 or 68 or 69 or 70 or 71 or 72 or 73 or 74 or 75 or 76 or 77 or 78 or 79 or 80 or 81 or 82 or 83 or 84 or 85 or 86 or 87 or 88 or 89 or 90 or 91 940432
93. 28 and 60 and 92 687

Supplementary Table S2: Extracted data from eligible studies

Reference	Cultivar	Year	Location	Iron content, µg/g	Other information
**Wheat**					
Dadswell, 1935	Comeback	1930	Wimmera, VIC	51	
Dadswell, 1935	Comeback	1930	Goulburn Valley, VIC	62	
Dadswell, 1935	Federation	1930	Wimmera, VIC	49	
Dadswell, 1935	Free gallipoli	1930	Central VIC	62	
Dadswell, 1935	Free gallipoli	1930	Central VIC	37	
Dadswell, 1935	Free gallipoli	1930	Goulburn Valley, VIC	41	
Dadswell, 1935	Free gallipoli	1930	Wimmera, VIC	51	
Dadswell, 1935	Free gallipoli	1930	Western VIC	44	
Dadswell, 1935	Major	1930	Goulburn Valley, VIC	45	
Dadswell, 1935	Major	1930	Central VIC	49	
Dadswell, 1935	Minister	1930	Goulburn Valley, VIC	58	
Dadswell, 1935	Nabawa	1930	Central VIC	46	
Dadswell, 1935	Nabawa	1930	Goulburn Valley, VIC	58	
Dadswell, 1935	Nizam	1930	Goulburn Valley, VIC	44	
Dadswell, 1935	Nizam	1930	Central VIC	46	
Dadswell, 1935	Nizam	1930	Goulburn Valley, VIC	46	
Dadswell, 1935	Nizam	1930	Wimmera, VIC	59	
Dadswell, 1935	Ranee	1930	Goulburn Valley, VIC	44	
Dadswell, 1935	Ranee	1930	Mallee, VIC	58	
Dadswell, 1935	Federation	1931	North Eastern, VIC	35	
Dadswell, 1935	Free gallipoli	1931	Central VIC	59	
Dadswell, 1935	Free gallipoli	1931	Mallee, VIC	34	
Dadswell, 1935	Free gallipoli	1931	North Eastern, VIC	45	
Dadswell, 1935	Ranee	1931	Mallee, VIC	32	
Dadswell, 1935	Sepoy	1931	Mallee, VIC	41	

Murphy and Law, 1974	Triticum aestivum, unspecified	1971-1972	QLD	54.3	Range 29-110, mean of 78 samples
Murphy and Law, 1974	Triticum aestivum, unspecified	1972-1973	VIC	43.2	Range 41-45.5, mean of 2 samples
Murphy and Law, 1974	Triticum aestivum, unspecified	1972-1973	WA	45	Range 47-49.1, mean of 2 samples
Murphy and Law, 1974	Triticum aestivum, unspecified	1972-1973	SA	45.8	Range 45.5-46.1, mean of 2 samples
Murphy and Law, 1974	Triticum aestivum, unspecified	1972-1973	NSW	54.5	Range 50-63, mean of 4 samples
Mugford and Steel, 1980	Triticum aestivum, unspecified	1977	TAS	36.8	Range: 36.5 - 37.3, from 3 mills
Mugford and Steel, 1980	Triticum aestivum, unspecified	1977	SA	38.3	Range: 34.8 - 44.3, from 12 mills
Mugford and Steel, 1980	Triticum aestivum, unspecified	1977	VIC	39.7	Range: 38.5 - 40.8, from 7 mills
Mugford and Steel, 1980	Triticum aestivum, unspecified	1977	NSW	42.9	Range: 37.6 - 47.4, from 24 mills
Mugford and Steel, 1980	Triticum aestivum, unspecified	1977	QLD	44.4	Range: 40.8 - 50.9, from 11 mills
Mugford and Steel, 1980	Triticum aestivum, unspecified	1977	WA	45.1	Range: 43.4 - 47.1, from 5 mills
Mugford and Steel, 1980	Triticum aestivum, unspecified	1977	Australia	41.8	Range: 34.8 - 50.9, from 62 mills
Zarcinas et al, 1987	Triticum aestivum, unspecified	1987	Australia, unknown location	60	Single sample
Bolland et al, 1993	Gutha	1987	South Carrabin, WA	37	SE 7
Bolland et al, 1993	Jacup	1987	Badgingarra, WA	83	SE 5
Bolland et al, 1993	Eruda	1987	Cadoux, WA	51	SE 2
Batten, 1994	Australian Hard	1987- 1990	Gladstone, Brisbane, Port Adelaide, Port Giles, Ardrossan, Wallaroo, Port Pirie, Port Lincoln, Thevenard, Albany, Fremantle, Geraldton	34.7	Range: 24.5 - 52.6, 14 samples

Batten, 1994	Australian Standard White	1987- 1990	Gladstone, Brisbane, Port Kembla, Portland, Geelong, Port Adelaide, Port Giles, Ardrossan, Wallaroo, Port Pirie, Thevenard, Esperance, Albany, Fremantle, Geraldton	38.2	Range: 23.6 - 69.4, 20 samples
Batten, 1994	Prime Hard	1987- 1990	Gladstone, Brisbane, Newcastle	38.7	Range: 30.1 - 52.95 samples
Batten, 1994	Soft wheat	1987- 1990	Albany, Fremantle, White Rose Flour Mills	36.8	Range: 31.9 - 52.6, 7 samples
Hocking, 1994	Egret	1994	Griffith, NSW	26	
Booth et al, 1996	Soft wheat	1993	Australia, multiple locations	35.4	Range: 30 - 40, 4 samples
Morrison, 1996	Australian Prime Hard	1990-1991	QLD	44.9	Range: 44.2 - 45.8
Morrison, 1996	Australian Prime Hard	1990-1991	NSW	41.8	Range: 25.3 - 52.2
Morrison, 1996	Australian Std white	1990-1991	QLD	40	Range: 36.4 - 43.5
Morrison, 1996	Australian Std white	1990-1991	NSW	28.6	Range: 19.9 - 39.2
Morrison, 1996	Australian Std white	1990-1991	SA	28.6	Range: 16.7 - 39.0
Morrison, 1996	Australian Std white	1990-1991	VIC	29.5	Range: 19.0 - 36.5
Morrison, 1996	General Purpose	1990-1991	QLD	45.2	Range: -
Morrison, 1996	General Purpose	1990-1991	NSW	29.7	Range: 21.3 - 42.1
Morrison, 1996	General Purpose	1990-1991	SA	31.1	Range: 23.7 - 37.5
Morrison, 1996	Australian Hard	1990-1991	QLD	42.1	Range:36.8 - 49.5
Morrison, 1996	Australian Hard	1990-1991	NSW	39.3	Range: 26.0 - 51.2
Morrison, 1996	Australian Hard	1990-1991	SA	29.2	Range: 26.0 - 35.5
Morrison, 1996	Australian Hard	1990-1991	VIC	30.8	Range: 26.7-38.6
Morrison, 1996	Australian Prime Hard	1991-1992	QLD	34.1	Range: 22.3 - 41.3

Morrison, 1996	Australian Prime Hard	1991-1992	NSW	33.9	Range: 28.8 - 39.2
Morrison, 1996	Australian Std white	1991-1992	QLD	31.1	Range: 27.7 - 34.4
Morrison, 1996	Australian Std white	1991-1992	NSW	26.7	Range: 20.8 - 34.8
Morrison, 1996	Australian Std white	1991-1992	SA	23.1	Range: 20.3 - 26.6
Morrison, 1996	Australian Std white	1991-1992	VIC	23.8	Range: 21.0 - 26.6
Morrison, 1996	Australian Std white	1991-1992	WA	26.1	Range: 23.0 - 30.3
Morrison, 1996	Australian Hard	1991-1992	QLD	35.3	Range: 33.3 - 37.2
Morrison, 1996	Australian Hard	1991-1992	NSW	31.9	Range: 27.1 - 43.8
Morrison, 1996	Australian Hard	1991-1992	SA	27.1	Range: 22.4 - 34.6
Morrison, 1996	Australian Hard	1991-1992	VIC	25.3	Range: 21.0 - 32.7
Morrison, 1996	Australian Hard	1991-1992	WA	28.8	Range: 28.1 - 29.5
Morrison, 1996	Australian Soft	1991-1992	WA	26.4	Range: 24.6 - 29.8
Morrison, 1996	General Purpose	1991-1992	QLD	33.7	Range: -
Morrison, 1996	General Purpose	1991-1992	SA	24.8	Range: 19.4 - 30.3
Morrison, 1996	General Purpose	1991-1992	WA	29.2	Range: 27.1 - 32.9
Morrison, 1996	Australian Prime Hard	1992-1993	QLD	31.6	Range: 27.8 - 35.2
Morrison, 1996	Australian Std white	1992-1993	QLD	29.2	Range: 28.4 - 30.1
Morrison, 1996	Australian Hard	1992-1993	QLD	31.1	Range: 29.5 - 32.6
Ryan et al, 2004	Vulcan	1991	Ardlethan, NSW	33	Mean of 2 plots
Ryan et al, 2004	Vulcan/Janz	1992	Ardlethan, NSW	19	Mean of 12 plots
Ryan et al, 2004	Janz	1993	Ardlethan, NSW	21	Mean of 15 plots
Ryan et al, 2004	Dollarbird	1993	Yenda, NSW	22	Mean of 15 plots
Fernando et al, 2012	Yitpi	2008	Walpeup, VIC	27.5	
Fernando et al, 2012	Yitpi	2009	Walpeup, VIC	40	
Norton, 2013	Yipti + Gladius	2008 - 2009	Lower EP, SA	30.2	SD 1.8

Norton, 2013	Yipti + Gladius	2008 - 2009	Yorke Peninsula, SA	31.3	SD 1.8
Norton, 2013	Yipti + Gladius	2008 - 2009	Mallee, VIC	34.9	SD 1.5
Norton, 2013	Yipti + Gladius	2008 - 2009	South East SA	35.9	SD 1.9
Norton, 2013	Yipti + Gladius	2008 - 2009	Upper EP, SA	35.9	SD 1.2
Norton, 2013	Yipti + Gladius	2008 - 2009	Wimmera, VIC	36.2	SD 1.9
Norton, 2013	Yipti + Gladius	2008 - 2009	Mid North SA	39.1	SD 1.6
Norton, 2013	Yipti + Gladius	2008 - 2009	South West NSW	39.5	SD 1.2
Norton, 2013	Yipti + Gladius	2008 - 2009	Murray Mallee, SA	40.1	SD 1.4
Norton, 2013	Yipti + Gladius	2008 - 2009	North Central VIC	41	SD 3.0
Norton, 2013	Yipti + Gladius	2008 - 2009	South East NSW	42.1	SD 2.2
Norton, 2013	Yipti + Gladius	2008 - 2009	North East VIC	44.3	SD 3.0
Fernando et al, 2014	Yitpi	2009	Horsham, VIC	41	Mean of 48 replicates
Fernando et al, 2014	Janz	2009	Horsham, VIC	38	Mean of 48 replicates
Ishida et al, 2014	Australian Prime Hard	2009	Eastern Australia	44.4	SD 7.4; mean of 29 samples
Ishida et al, 2014	Australian Standard White	2009 - 2010	WA and Eastern Australia	36.5	SD 5.1; mean of 59 samples
Broom et al, 2014	Australian Prime Hard	2012	Australia, unspecified	42	Single sample
Rose et al, 2015	Wyalcatchem	2012	Lismore, NSW	63.1	Mean of 3 replicates
Jin et al, 2019	Yipti	2010	VIC, Chromosol soil	66.4	Mean of 4 replicates
Jin et al, 2019	Yipti	2010	VIC, Vertisol soil	77.6	Mean of 4 replicates
Jin et al, 2019	Yipti	2010	VIC, Calcarosol soil	85	Mean of 4 replicates
Beasley et al, 2019	Triticum aestivum, unspecified	2015	Merredin and Katanning, WA	40	Mean of 7 replicates
Joukhadar et al, 2021	Triticum aestivum, unspecified	2017	Narrabri, NSW	39.8	SD 5.1; mean of 200 genotype samples
Joukhadar et al, 2021	Triticum aestivum, unspecified	2018	Merredin, WA	37.1	SD 4.7; mean of 200 genotype samples
Joukhadar et al, 2021	Triticum aestivum, unspecified	2018	Horsham, VIC	39.1	SD 5.6; mean of 200 genotype samples

Rice

Wills et al, 1982	Calrose, medium grain	1982	NSW	11	Composite sample of 4 packets of Sunbrown rice, bought in Sydney
Wills et al, 1982	Starbonnet, long grain	1982	QLD	13	Composite sample of 4 packets of Mahatma bought in Sydney
Marr et al, 1995	Amaroo, (medium grain)	1992	Albury, NSW	13	Range: 5-67, 90 samples
Booth et al, 1996	Oryza sativa, unspecified	1993	Australia, multiple locations	12.3	Range: 11.5-13; mean of 7 samples
Marr et al, 1999	Amaroo (medium grain)	1992 - 1993	Yanco, NSW	25	Mean of 36 samples
Marr et al, 1999	YRL38 (long grain)	1992 - 1993	Yanco, NSW	25	Mean of 36 samples
Marr et al, 1999	Langi (long grain)	1992 - 1993	Yanco, NSW	28	Mean of 36 samples
Marr et al, 1999	Amaroo (medium grain)	1993 - 1994	Yanco, NSW	26.1	Mean of 20 samples
Marr et al, 1999	Pelde (long grain)	1993 - 1994	Yanco, NSW	23.9	Mean of 20 samples
Marr et al, 1999	Langi (long grain)	1993 - 1994	Yanco, NSW	24.2	Mean of 20 samples
Marr et al, 1999	Kyeema (long grain)	1993 - 1994	Yanco, NSW	22.2	Mean of 20 samples
Wurm et al, 2012	Oryza sativa, unspecified	2008	Australia, multiple locations	11	Mean of 'large number of pooled samples'
Broom et al, 2014	Oryza sativa, unspecified	2012	Australia, unspecified	8	Single sample
Birch et al, 2023	Oryza sativa, unspecified	2020	Australia, unspecified	26	Triplicate sample
Rahman and Naidu, 2023	Oryza sativa, unspecified (medium grain)	2023	Australia, unspecified	11.5	Triplicate sample
